# MicroRNA-34a Promotes EMT and Liver Fibrosis in Primary Biliary Cholangitis by Regulating TGF-*β*1/smad Pathway

**DOI:** 10.1155/2021/6890423

**Published:** 2021-04-23

**Authors:** Ying Pan, Jing Wang, Lan He, Fengchun Zhang

**Affiliations:** ^1^Department of Rheumatology and Immunology, First Affiliated Hospital of Xi'an Jiaotong University, Xi'an 710061, China; ^2^Department of Rheumatology, Peking Union Medical College Hospital, Chinese Academy of Medical Sciences and Peking Union Medical College, Beijing 100730, China

## Abstract

**Background and Aims:**

Primary biliary cholangitis (PBC) is an autoimmune cholestatic liver disease. We found microRNA-34a (miR-34a), as the downstream gene of p53, was overexpressed in some of fibrogenic diseases. In this study, we sought to explore whether miR-34a plays a role in the fibrosis of PBC.

**Methods:**

The peripheral blood of PBC patients and controls was collected to analyze the level of miR-34a. Human intrahepatic biliary epithelial cells (HIBEC) were cultured. The expression of miR-34a was regulated by miR-34a mimics and inhibitor. The biomarkers of epithelium-mesenchymal transition (EMT), fibrogenesis, inflammation, and transforming growth factor- (TGF-) *β*1/smad pathway were analyzed.

**Results:**

We found that miR-34a was overexpressed in the peripheral blood in PBC patients. In vitro, overexpressed miR-34a increased the EMT and fibrogenesis activity of HIBEC. Transforming growth factor-beta type 1 receptor (T*β*R1), TGF-*β*1, and p-smad2/3 were upregulated by miR-34a. Inflammatory factors such as IL-6 and IL-17 were also upregulated. Finally, we showed that miR-34a promoted EMT and liver fibrosis in PBC by targeting the TGF-*β*1/smad pathway antagonist transforming growth factor-beta-induced factor homeobox 2 (TGIF2).

**Conclusions:**

Our findings show that miR-34a plays an important role in the EMT and fibrosis of PBC through the TGF-*β*1/smad pathway by targeting TGIF2. This study suggests that miR-34a may be a new marker of fibrogenesis in PBC. Inhibition of miR-34a may be a promising strategy in treating PBC and improving the prognosis of the disease.

## 1. Introduction

Primary biliary cholangitis (PBC) is a progressive autoimmune cholestatic liver disease. In PBC, the bile ducts are infiltrated by immune and inflammatory cells causing structural damage, ductopenia, and peribiliary stromal expansion. The function of hepatic stellate cell (HSC) in liver fibrosis is the most widely investigated. However, the simple paradigm that fibrosis and tissue scarring are only due to the activation and progressive hyperplasia of HSC does not fully satisfy the features of biliary fibrosis [1]. Recently, more and more evidence shows that profibrogenic myofibroblasts are a heterogeneous population of cells and are not derived entirely from quiescent HSC [2]. It has been suggested that cholangiocytes may contribute to the hepatic fibrogenic cell population by undergoing epithelial–mesenchymal transition (EMT) [3–5]. Immunohistochemistry studies of liver sections from patients with PBC showed attenuated epithelial markers and increased mesenchymal markers in cholangiocytes. Prevention of the development of EMT may control and even reverse liver fibrosis [6]. Chronic inflammation of the bile ducts can induce biliary epithelial cell (BEC) proliferation, which can eventually progress to biliary fibrosis. However, the role of EMT in BEC is still controversial [7]. In one study of a PBC patient after transplantation identified BEC EMT as a key pathogenetic process. EMT occurs before the development of any other features of recurrent PBC, suggesting EMT may be an initiating event (potentially explaining BEC loss) and is driven by TGF-*β* [8]. TGF-*β*1 is generally considered one of the master positive regulators of EMT [9].

MicroRNAs (miRNAs) are functional small noncoding RNA molecules that posttranscriptionally affect mRNA stability and translation by targeting the 3′-untranslated region (3′-UTR) of various transcripts [10]. Many different types of miRNAs are involved in different stages of chronic liver diseases, including liver fibrosis [11]. Hundreds of miRNAs differentially expressed in the sera and peripheral blood mononuclear cells (PBMCs) of PBC patients [12]. The miR-34a family was first discovered as the downstream gene of p53 [13]. Interestingly, a role of miR-34a in the inhibition of EMT was described in cancer cell lines [14–16]. However, miR-34a was also found to induce the fibrosis in myocardial infarction [17] and fibrotic kidney [18].

In this study, we analyzed the level of miR-34a in peripheral blood in PBC patients. The function of miR-34a in EMT and fibrosis of human intrahepatic biliary epithelial cells (HIBEC) through the TGF-*β*1/smad pathway was studied.

## 2. Materials and Methods

### 2.1. Patients

We analyzed the serum miR-34a of 30 PBC patients and 30 controls. All patients were inpatients from the Rheumatology and Immunology Department of the First Affiliated Hospital of Xi'an Jiaotong University. All of them fulfilled the diagnosis of PBC in the European Association for the Study of the Liver (EASL) clinical practice guidelines [19]. All participating patients signed consent forms before study enrollment. The study protocols were performed in accordance with the Declaration of Helsinki and the guidelines for good medical practice in China.

### 2.2. Cell Culture

HIBEC (Jennio Biotech Co. Ltd., Guangzhou) were cultured in RPMI-1640 containing 10% fetal bovine serum, 1% penicillin G, and streptomycin solution under standard condition (37°C in 5% CO_2_). Cells were passaged when the cells reached 80% confluence, and passage 2-3 was used in this study.

### 2.3. miR-34a Mimics and Inhibitor Transfection and Cell Proliferation Assay

For transfection, miR-34a mimics, miR-34a inhibitor, and negative controls (Genepharma Co. Shanghai) were transfected using lipofectamine 2000 (Invitrogen, US) according to the manufacturer's protocol. The sequences of miR-34a mimics, miR-34a inhibitor, and controls were listed in Supplementary Table 1. We measured the proliferation activity of HIBEC by using cell counting kit-8 (CCK8, Biosharp Co., Guangzhou). 10 μl CCK8 was added to each well. The absorbance was measured. The results were shown as optical density measured at 450 nm.

### 2.4. Immune Cytochemistry Assay

Immune cytochemistry assay was carried out according to the standard protocol. Primary antibodies were added to incubate HIBEC cells transfected by miR-34a mimics, inhibitors, and controls overnight at 4°C. Fluorochrome-conjugated secondary antibodies were applied to the cells for 1 hour at 37°C in dark. Images were observed and collected under a fluorescence microscope (Olympus BX53 microscope, Olympus Corporation, Japan). Details of the antibodies were listed in Supplementary Table 2.

### 2.5. Ribonucleic Acid (RNA) Extracts and Quantitative Real-Time Polymerase Chain Reaction (qRT-PCR)

Total RNA was extracted from cultured HIBECs by TRIzol reagent (Aidlab, Beijing) according to the manufacturer's protocol. qRT-PCR was performed by EDC-810 PCR system (Eastwin Biotechnology, Beijing) and the SYBR Green Master Mix (Vazyme Biotech Co., Nanjing). Primers used for qRT-PCR were listed in Supplementary Table 3.

### 2.6. Western Blotting Analysis

Western blotting was carried out according to the standard protocol. Protein was extracted from the HIBECs with lysis buffer and quantified by the bicinchoninic acid method (Beyotime Biotechnology, Shanghai). Primary antibodies were applied to membranes overnight at 4°C. After that, horseradish peroxidase- (HRP-) conjugated secondary antibodies were applied to the membranes for 2 hours at 37°C. Bound antibodies were visualized with enhanced chemiluminescence (ECL, Thermo, US) and quantified by using Glyko BandScan software version 4.0. Details of the antibodies were listed in Supplementary Table 4.

### 2.7. 3′-UTR Reporter Construct, Transfection, and Dual-Luciferase Reporter Assay

To determine how miR-34a acted on the TGF-*β*1/smad pathway, we predicted miRNA target genes by searching TargetScan (http://www.targetscan.org), miRanda (http://www.microrna.org), and miRBase (http://www.mirbase.org). We noticed that the transforming growth factor-beta-induced factor homeobox 2 (TGIF2) gene, which was known as a modulation factor in the TGF-*β*1/smad pathway [20], was probable the target gene of miR-34a.

**Figure 1:**
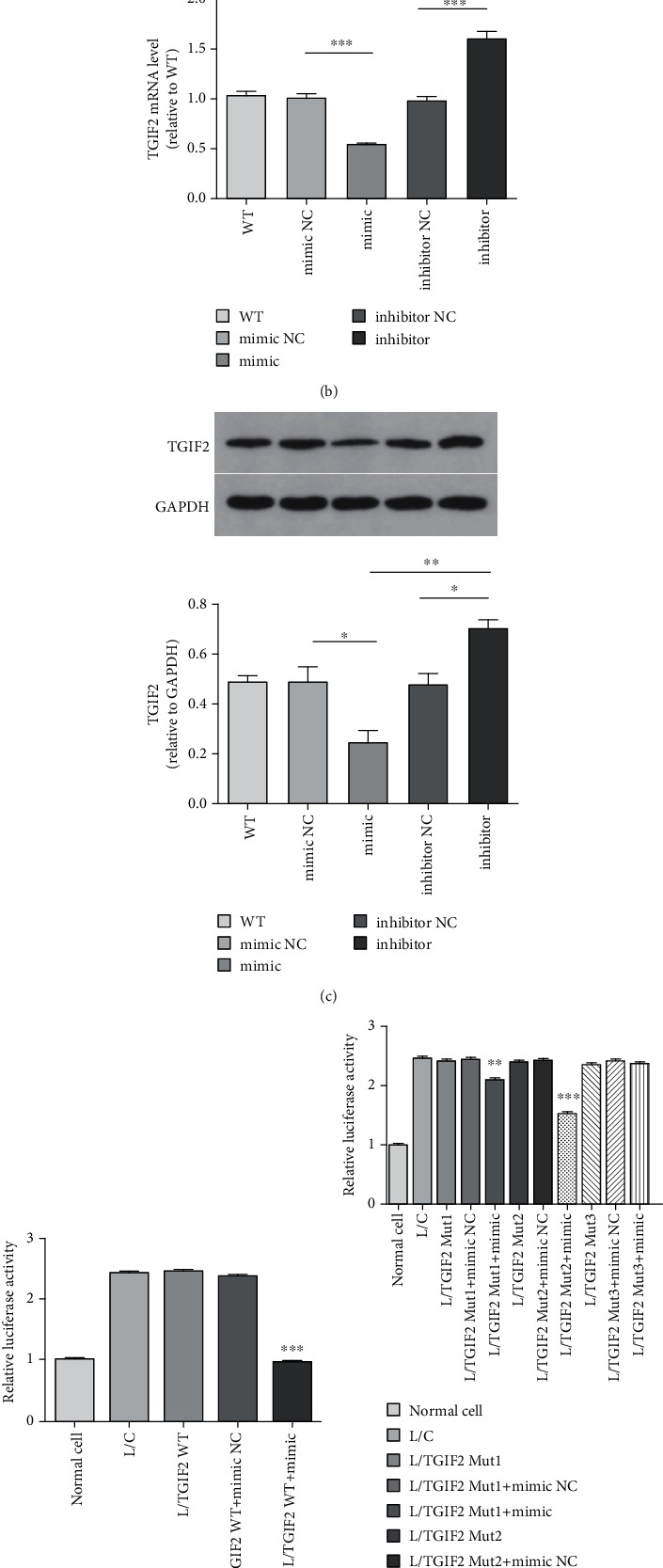
TGIF2 was the target gene of miR-34a. (a) The predicted two binding sequence of miR-34a on 3′-UTR of TGIF2 and their mutations. (b) TGIF2 mRNA level (miR-34a mimics vs. NC, *P* < 0.0001^∗∗∗^; miR-34a mimics vs. inhibitor, *P* < 0.0001^∗∗∗^; and miR-34a inhibitor vs. NC, *P* < 0.0001^∗∗∗^). (c) Relative TGIF2 protein expression (miR-34a mimics vs. NC, *P* = 0.0334^∗^; miR-34a mimics vs. inhibitor, *P* = 0.0015^∗∗^; and miR-34a inhibitor vs. NC, *P* = 0.0184^∗^). (d) Relative luciferase activity of wild-type TGIF2 (L/TGIF2 WT+mimics vs. L/TGIF2 WT+NC, *P* < 0.0001^∗∗∗^ and L/TGIF2 WT+mimics vs. L/TGIF2 WT, *P* < 0.0001^∗∗∗^). (e) Relative luciferase activity of three types mutations of TGIF2 (L/TGIF2 mut1+mimics vs. L/TGIF2 mut1+NC, *P* = 0.0026^∗∗^; L/TGIF2 mut2+mimics vs. L/TGIF2 mut2+NC, *P* < 0.0001^∗∗∗^; and L/TGIF2 mut3+mimics vs. L/TGIF2 mut3+NC, ns).

The wide type (WT) of human TGIF2 3′-UTR containing miR-34a binding sites was PCR amplified from human cDNA library. Mutated TGIF2 3′-UTR, voiding miR-34a binding, was generated by a Site-Directed Muta-genesis Kit (Biofavor biotech design, China). 3′-UTR of TGIF2 had two binding sites of miR-34a, one was 90-97 and the other was 608-615 (Figure 1(a)). To confirm the binding site of TGIF2, we constructed three kinds of TGIF2 mutations, L/TGIF2 Mut1 (mutation at 90-97), L/TGIF2 Mut2 (mutation at 608-615), and L/TGIF2 Mut3 (mutations of both two sites) (Figure 1(a)). The HEK-293 cell line has high transfection efficiency and stable expression of proteins. Therefore, we used HEK-293 cell line to verify whether TGIF2 was truly targeted by miR-34a. HEK-293 cells were seeded in 12-well format dishes at 2 × 10^5^/well and transfected with one of the five luciferase reporter vectors, L/TGIF2 WT, L/TGIF2 Mut1, L/TGIF2 Mut2, L/TGIF2 Mut3, and L/C (control), along with miR-34a mimics or negative control (NC). Relative luciferase activities were measured at 24 hours after transfection by a dual-luciferase reporter assay system (Beyotime Biotechnology, Shanghai) according to the manufacturer's protocol.

### 2.8. TGIF2 siRNA Interference Assay

**Figure 2:**
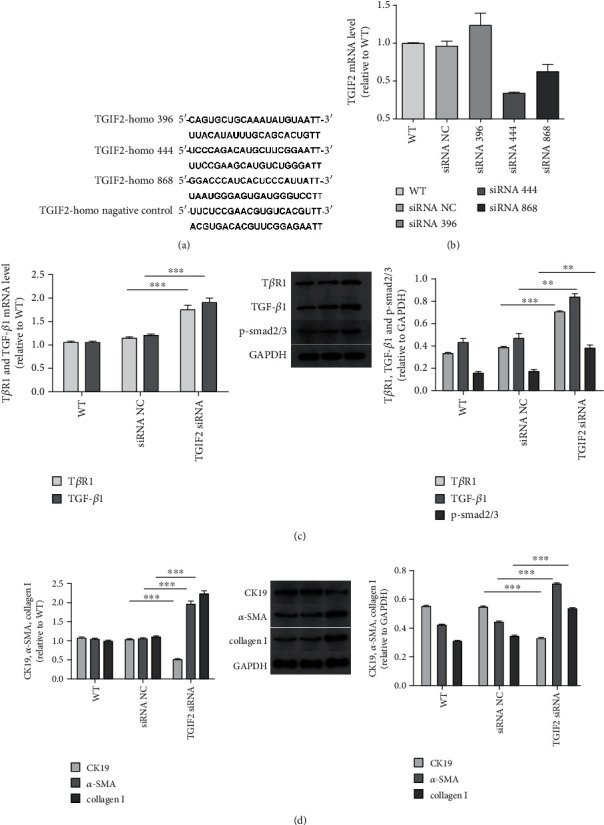
TGIF2 interacted with the TGF-*β*1/smad pathway. (a) Sequences of three TGIF2 siRNAs and negative control. (b) TGIF2 mRNA level after transfected by siRNAs. (c) T*β*R1 and TGF-*β*1 mRNA levels (for T*β*R1, TGIF2 siRNA vs. NC, *P* < 0.0001^∗∗∗^; for TGF-*β*1, TGIF2 siRNA vs. siRNA NC, *P* < 0.0001^∗∗∗^). Relative T*β*R1, TGF-*β*1, and p-smad2/3 protein expressions (for T*β*R1, TGIF2 siRNA vs. NC, *P* < 0.0001^∗∗∗^; for TGF-*β*1, TGIF2 siRNA vs. NC, *P* = 0.0047^∗∗^; and for p-smad2/3, TGIF2 siRNA vs. NC, *P* = 0.0039^∗∗^). (d) CK19, *α*-SMA, and collagen I mRNA levels between TGIF2 siRNA and NC (*P* < 0.0001^∗∗∗^, *P* < 0.0001^∗∗∗^, and *P* < 0.0001^∗∗∗^). Relative CK19, *α*-SMA, and collagen I protein expressions between TGIF2 siRNA and NC (*P* < 0.0001^∗∗∗^, *P* = 0.0002^∗∗∗^, and *P* = 0.0004^∗∗∗^).

To verify whether TGIF2 was truly interacting with the TGF-*β*1/smad pathway, we designed the TGIF2 interference assay. Three different siRNAs were designed (Genepharma Co., Shanghai). The sequences of siRNAs were shown in Figure 2(a). We chose the siRNA which had the highest interference efficiency to complete the following tests.

### 2.9. Statistical Analysis

All data were presented as mean ± standard deviation (SD) of the mean for at least three biological repeats. Differences between the groups were analyzed using Student's *t*-test by SPSS 22.0 software (IBM SPSS Collaboration, US). The differences were considered statistically significant if *P* < 0.05.

## 3. Results

### 3.1. miR-34a Was Overexpressed in PBC Patients

**Figure 3:**
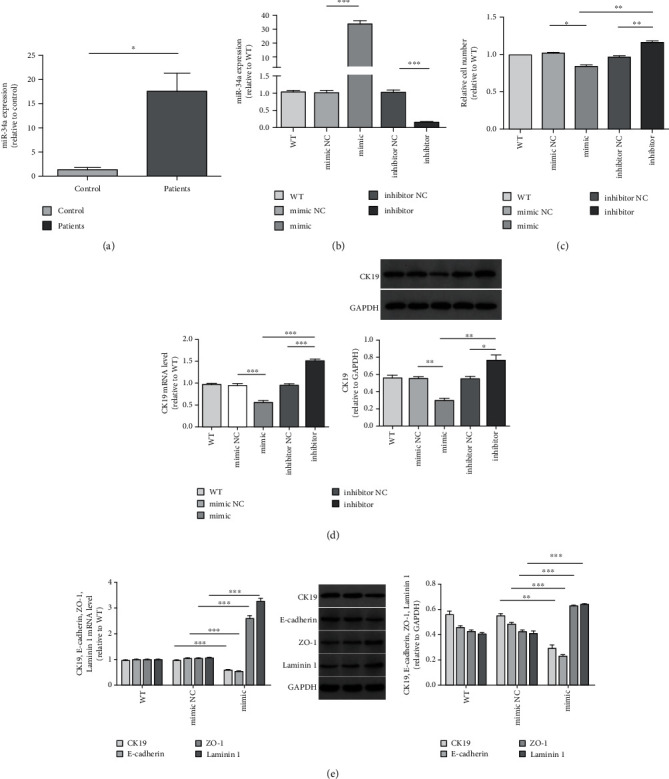
miR-34 expression in the serum of PBC patients and healthy controls and HIBEC proliferation in vitro. (a) miR-34a was significantly overexpressed in the sera of PBC patients (*P* = 0.048^∗^). (b) Expression of miR-34a of HIBEC transfected by miR-34a mimics, inhibitor, and controls (miR-34a mimics vs. NC, *P* < 0.0001^∗∗∗^ and miR-34a inhibitor vs. NC, *P* < 0.0001^∗∗∗^). (c) Relative cell number measured by using CCK8 (miR-34a mimics vs. NC, *P* = 0.0155^∗^; miR-34a mimics vs. inhibitor, *P* = 0.0039^∗∗^; and miR-34a inhibitor vs. NC, *P* = 0.0037^∗∗^). (d) CK19 mRNA level of each group (miR-34a mimics vs. NC, *P* < 0.0001^∗∗∗^; miR-34a mimics vs. inhibitor, *P* < 0.0001^∗∗∗^; and miR-34a inhibitor vs. NC, *P* < 0.0001^∗∗∗^). Relative CK19 protein expression (miR-34a mimics vs. NC, *P* = 0.0013^∗∗^; miR-34a mimics vs. inhibitor, *P* = 0.0026^∗∗^; and miR-34a inhibitor vs. NC, *P* = 0.0404^∗^). (e) CK19, E-cadherin, ZO-1, and laminin 1 mRNA levels between miR-34a mimics and NC (*P* < 0.0001^∗∗∗^, *P* < 0.0001^∗∗∗^, *P* < 0.0001^∗∗∗^, and *P* < 0.0001^∗∗∗^). CK19, E-cadherin, ZO-1, and laminin 1 protein expressions between miR-34a mimics and NC (*P* = 0.0013^∗∗^, *P* = 0.0003^∗∗∗^, *P* = 0.0007^∗∗∗^, and *P* = 0.0003^∗∗^).

We found that miR-34a was significantly overexpressed in the serum of PBC patients (Figure 3(a)).

### 3.2. miR-34a Inhibited the HIBEC Proliferation and Induced EMT of HIBEC

**Figure 4:**
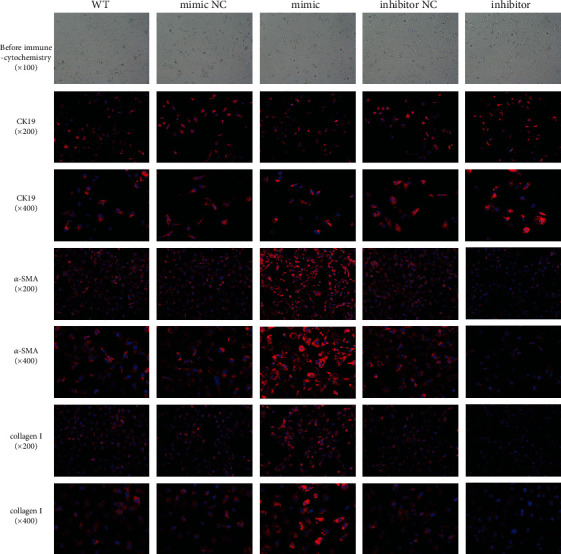
Cell morphology and the expression of CK19, a-SMA, and collagen I by immune cytochemistry after transfection with miR-34a mimics, inhibitor, and controls. Before immune cytochemistry assay, cell proliferation was inhibited by miR-34a. By immune cytochemistry, we found that miR-34a downregulated CK19 and upregulated *α*-SMA and collagen I.

We used miR-34a mimics and inhibitor to regulate the expression of miR-34a in HIBEC. After 48 h of transfection, HIBEC transfected by miR-34a mimics expressed the highest level of miR-34a (Figure 3(b)). By measuring the absorbance at 450 nm in CCK8 assay, we found that miR-34a upregulation inhibited the proliferation of HIBEC, while miR-34a downregulation promoted the proliferation of HIBEC (Figure 3(c)). We observed the consistent result by cell morphology before immune cytochemistry assay (Figure 4).

**Figure 5:**
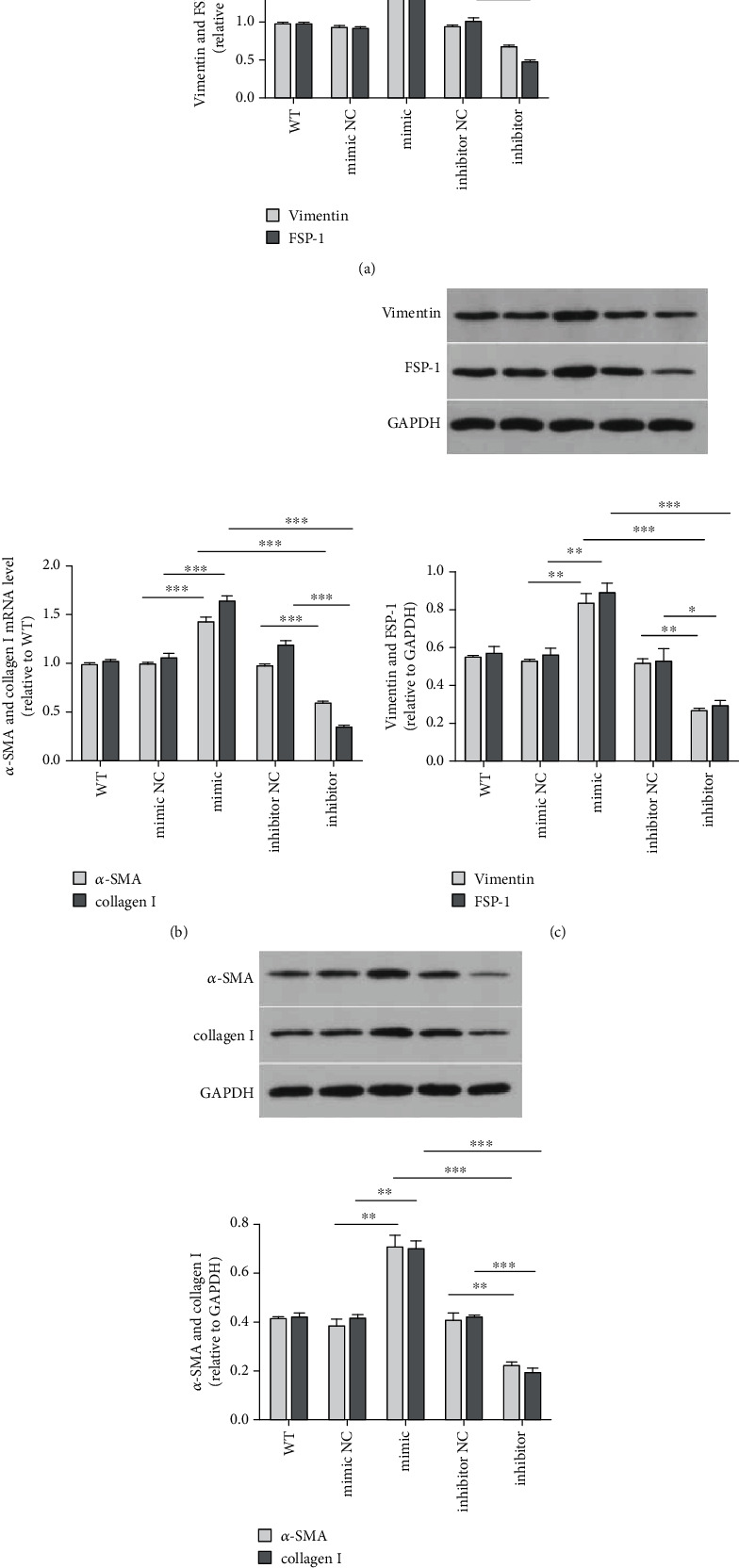
miR-34a induced EMT and fibrogenesis of HIBEC. (a) Vimentin and FSP-1 mRNA levels (for vimentin, miR-34a mimics vs. NC, *P* < 0.0001^∗∗∗^; miR-34a mimics vs. inhibitor, *P* < 0.0001^∗∗∗^, and miR-34a inhibitor vs. NC, *P* < 0.0001^∗∗∗^; for FSP-1, miR-34a mimics vs. NC, *P* < 0.0001^∗∗∗^; miR-34a mimic vs. inhibitor, *P* < 0.0001^∗∗∗^; and miR-34a inhibitor vs. NC, *P* < 0.0001^∗∗∗^). (b) *α*-SMA and collagen I mRNA levels (for *α*-SMA, miR-34a mimics vs. NC, *P* < 0.0001^∗∗∗^; miR-34a mimics vs. inhibitor, *P* < 0.0001^∗∗∗^; and miR-34a inhibitor vs. NC, *P* < 0.0001^∗∗∗^; for collagen I, miR-34a mimics vs. NC, *P* < 0.0001^∗∗∗^; miR-34a mimics vs. inhibitor, *P* < 0.0001^∗∗∗^; and miR-34a inhibitor vs. NC, *P* < 0.0001^∗∗∗^). (c) Relative vimentin and FSP-1 protein expressions (for vimentin, miR-34a mimics vs. NC, *P* = 0.0031^∗∗^; miR-34a mimics vs. inhibitor, *P* = 0.0003^∗∗∗^; and miR-34a inhibitor vs. NC, *P* = 0.0015^∗∗^; for FSP-1, miR-34a mimics vs. NC, *P* = 0.0054^∗∗^; miR-34a mimics vs. inhibitor, *P* = 0.0004^∗∗∗^; and miR-34a inhibitor vs. NC, *P* = 0.0271^∗^). (d) Relative *α*-SMA and collagen I protein expressions (for *α*-SMA, miR-34a mimics vs. NC, *P* = 0.0047^∗∗^; miR-34a mimics vs. inhibitor, *P* = 0.0007^∗∗∗^; and miR-34a inhibitor vs. NC, *P* = 0.0083^∗∗^; for collagen I, miR-34a mimics vs. NC, *P* = 0.0023^∗∗^; miR-34a mimics vs. inhibitor, *P* = 0.0003^∗∗∗^, and miR-34a inhibitor vs. NC, *P* = 0.0006^∗∗∗^).

In addition, we analyzed the expression of cytokeratin 19 (CK19). miR-34a upregulation inhibited while miR-34a downregulation promoted CK19 expression in HIBEC (Figures 3(d) and 4). miR-34a had similar effects on E-cadherin as on CK19 (Figure 3(e)). We also found that miR-34a upregulation increased the expression of zonula occluden-1 (ZO-1), laminin 1 (Figure 3(e)), vimentin, and fibroblast-specific protein-1 (FSP-1) (Figures 5(a) and 5(c)). The result confirmed that miR-34a induced EMT of HIBEC.

CK19 was the specific biomarker of HIBEC. The decrease of CK19 might be caused by the declined number of HIBECs and EMT together. In CCK8 assay, the relative cell number of HIBEC transfected with miR-34a mimics decreased by 20.5%, compared to mimics NC (82.77% and 103.27%, respectively, Figure 3(c)). The relative CK19 mRNA in HIBEC transfected by miR-34a mimics was 0.5543 ± 0.03266, while it was 0.9392 ± 0.03893 in mimics NC (Figure 3(d)). So, CK19 mRNA decreased by 40.98%. Relative CK19 protein in HIBEC transfected by miR-34a mimics and NC was 0.2967 ± 0.02341 and 0.5487 ± 0.02118, respectively (Figure 3(d)). So, CK19 protein decreased by 45.93%. CK19 mRNA and protein decreased about twice than relative cell number decline (20.5%). The results showed EMT also inhibited the expression of CK19.

### 3.3. miR-34a Promoted HIBEC Fibrogenesis

We analyzed the expression of *α*-SMA and collagen I, which are biomarkers of fibrogenesis. We found that miR-34a upregulated the expression of *α*-SMA and collagen I (Figures 4, 5(b), and 5(d)). This result showed that miR-34a promoted fibrogenesis of HIBEC.

### 3.4. miR-34a Interacted with TGF-*β*1/smad Pathway

**Figure 6:**
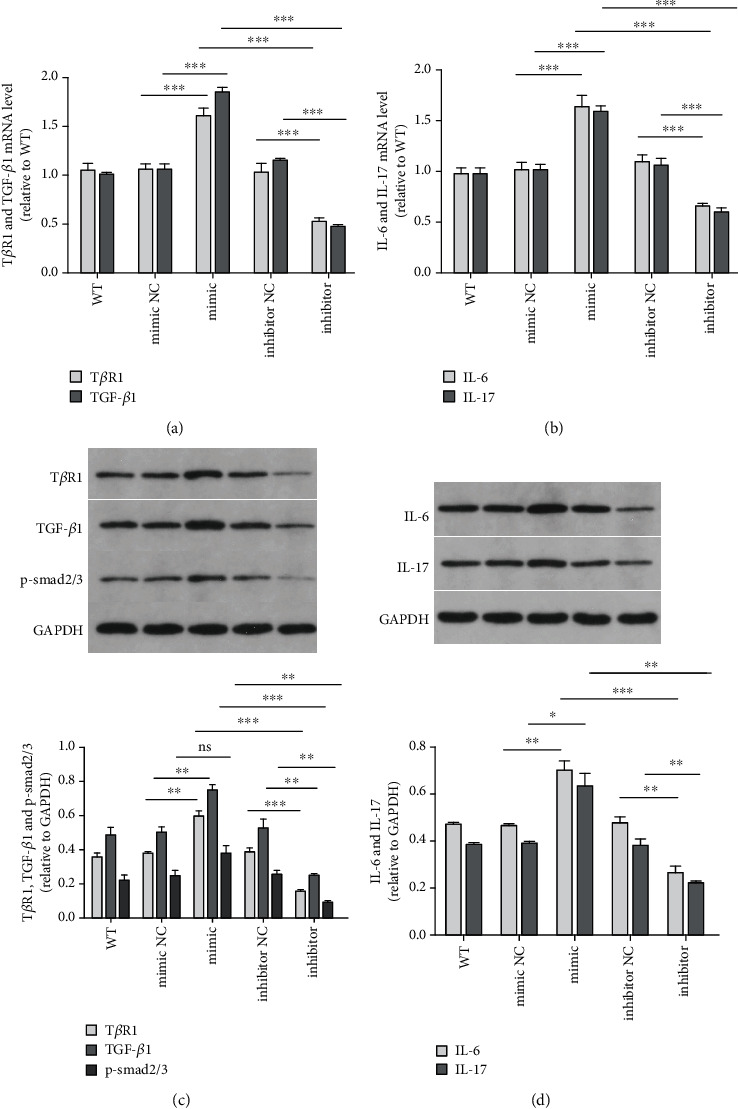
miR-34a promoted EMT and fibrosis of HIBEC through the TGF-*β*1/smad pathway and miR-34 upregulated inflammatory factors. (a) T*β*R1 and TGF-*β*1 mRNA levels (for T*β*R1, miR-34a mimics vs. NC, *P* < 0.0001^∗∗∗^; miR-34a mimics vs. inhibitor, *P* < 0.0001^∗∗∗^; and miR-34a inhibitor vs. NC, *P* < 0.0001^∗∗∗^; for TGF-*β*1, miR-34a mimics vs. NC, *P* < 0.0001^∗∗∗^; miR-34a mimics vs. inhibitor, *P* < 0.0001^∗∗∗^; and miR-34a inhibitor vs. NC, *P* < 0.0001^∗∗∗^). (b) IL-6 and IL-17 mRNA levels (for IL-6, miR-34a mimics vs. NC, *P* = 0.0002^∗∗∗^; miR-34a mimics vs. inhibitor, *P* < 0.0001^∗∗∗^; and miR-34a inhibitor vs. NC, *P* < 0.0001^∗∗∗^; for IL-17, miR-34a mimics vs. NC, *P* = 0.0002^∗∗∗^, miR-34a mimics vs. inhibitor, *P* < 0.0001^∗∗∗^; and miR-34a inhibitor vs. inhibitor-NC, *P* < 0.0001^∗∗∗^). (c) Relative T*β*R1, TGF-*β*1, and p-smad2/3 protein expressions (for T*β*R1, miR-34a mimics vs. NC, *P* = 0.0013^∗∗^; miR-34a mimics vs. inhibitor, *P* < 0.0001^∗∗∗^; and miR-34a inhibitor vs. NC, *P* = 0.0006^∗∗∗^; for TGF-*β*1, miR-34a mimics vs. NC, *P* = 0.0039^∗∗^; miR-34a mimics vs. inhibitor, *P* < 0.0001^∗∗∗^; and miR-34a inhibitor vs. NC, *P* = 0.0090^∗∗^; for p-smad2/3, miR-34a mimics vs. NC, *P* = 0.0679, ns; miR-34a mimics vs. inhibitor, *P* = 0.0027^∗∗^, and miR-34a inhibitor vs. NC, *P* = 0.0041^∗∗^). (d) Relative IL-6 and IL-17 protein expressions (for IL-6, miR-34a mimics vs. NC, *P* = 0.004^∗∗^; miR-34a mimics vs. inhibitor, *P* = 0.0008^∗∗∗^; and miR-34a inhibitor vs. NC, *P* = 0.0047^∗∗^; for IL-17, miR-34a mimics vs. NC, *P* = 0.0186^∗^; miR-34a mimics vs. inhibitor, *P* = 0.0028^∗∗^; and miR-34a inhibitor vs. NC, *P* = 0.0062^∗∗^).

We found that miR-34a had the function on both EMT and fibrogenesis. The TGF-*β*1/smad pathway promotes fibrosis significantly and plays an important role in EMT. So, we next investigated whether the role of miR-34a in modulating EMT and fibrogenesis was through the TGF-*β*1/smad pathway. Upregulation of miR-34a increased the expression of T*β*R1, TGF-*β*1, and p-smad2/3 (Figures 6(a) and 6(c)). These data suggested that miR-34a promoted EMT and fibrogenesis through the TGF-*β*1/smad pathway. Also, inflammatory factors IL-6 and IL-17 were upregulated by miR-34a (Figures 6(b) and 6(d)).

### 3.5. TGIF2 Was the Target Gene of miR-34a

In order to verify if TGIF2 was truly targeted by miR-34a, we conducted a dual-luciferase reporter assay in HEK-293 cells.

miR-34a downregulated the expression of TGIF2 (Figures 1(b) and 1(c)). HEK-293 cells transfected with L/TGIF2 WT and miR-34a mimics had similar relative luciferase activity as normal cells (Figure 1(d)). The result showed TGIF2 was the target gene of miR-34a. When HEK-293 cells transfected with L/TGIF2 Mut1 and L/TGIF2 Mut2 together with miR-34a mimics, relative luciferase activities were significantly different from that of control groups, especially in group L/TGIF2 Mut2 together with miR-34a mimics (Figure 1(e)). When HEK-293 cells transfected with L/TGIF2 Mut3 and miR-34a mimics, the relative luciferase activity showed no difference from the control group. These results demonstrated that the 3′-UTR binding site of TGIF2 was at 90-97 and 608-615, while 90-97 played a more important role in the combination with miR-34a.

### 3.6. TGIF2 Interacted with TGF-*β*1/smad Pathway

In order to verify TGIF2 was targeted by miR-34a and then interacted with the TGF-*β*1/smad pathway, we designed the TGIF2 interference assay. Three different siRNAs were designed (Figure 2(a)). We tested the mRNA of TGIF2 after transfection with siRNAs (Figure 2(b)). We chose siRNA-2 (TGIF2-homo-444), which had the highest interference efficiency of 66.3%, to complete the following tests. Transfection of TGIF2 siRNA resulted in significant increased expression of T*β*R1, TGF-*β*1, and p-smad2/3 (Figure 2(c)). After transfection by TGIF2 siRNA, CK19 was downregulated, meanwhile, *α*-SMA and collagen I were upregulated (Figure 2(d)). The results showed that miR-34a promoted EMT and fibrogenesis of HIBEC through the TGF-*β*1/smad pathway by targeting TGIF2.

## 4. Discussion

In the present study, we identified the role of miR-34a in PBC. We found that HIBEC occurred in EMT when transfected with miR-34a mimics. EMT was characterized by decreased CK19, E-cadherin, and concomitant acquisition of mesenchymal markers, ZO-1, laminin 1, vimentin, and FSP-1.

CCK8 decreased while transfected with miR-34a mimics. This result demonstrated that miR-34a inhibited the proliferation of HIBEC. Wang et al. found miR-34a inhibited proliferation in esophageal squamous cell carcinoma by targeting LEF1 and inactivation of the Hippo-YAP1/TAZ signaling pathway [21]. And Tao et al. found miR-34a inhibited proliferation and promoted apoptosis of rat osteoarthritic cartilage cells thought the PI3K/Akt pathway [22]. Maybe miR-34a regulates HIBEC proliferation through other pathways, and this need further research to confirm.

*α*-SMA and collagen I were also upregulated by miR-34a. It showed that miR-34a promoted EMT and fibrogenesis of HIBEC. We confirmed that miR-34a promoted the expression of T*β*R1, TGF-*β*1, and p-smad2/3. This means miR-34a enhances the expression of the TGF-*β*1/smad pathway. Many studies showed that TGF-*β*1 mediated EMT in neoplastic diseases [23–26]. While in this study, we found TGF-*β*1 mediated EMT in HIBEC, which was not a tumor cell line. TGF-*β*1 also plays an important role in fibrogenic diseases [27–30]. Immunohistochemistry staining showed that TGF-*β*1 localized in the cytoplasm of hepatic cells, and liver sections from PBC patients showed higher levels of TGF-*β*1 than controls, showing the importance of TGF-*β*1 in the microenvironment of the disease involving the liver [31]. In another study of PBC mouse, TGF-*β*1 had a high concentration in the portal area of PBC patients [32]. Our study found PBC patients overexpressed miR-34a in sera. miR-34a may play an important role in the development of PBC through the TGF-*β*1/smad pathway.

miR-34a downregulated the expression of TGIF2. This result suggested that miR-34a interacted with TGIF2. Transforming growth factor-beta-induced factor (TGIF) is a transcriptional repressor. TGIF2 and TGIF have very similar DNA-binding homeodomains, and TGIF2 represses transcription when bound to DNA via a TGIF binding site. TGIF2 interacts with TGF-*β*-activated smads and represses TGF-*β*-responsive transcription [20]. smad proteins are important ligands for the TGF-*β* pathway to mediate intracellular signaling [33]. TGF-*β*1 signaling is initiated by binding to the type II receptor. Subsequently, this receptor dimerizes with its type I receptor and binds smad2 and smad3. This complex becomes phosphorylated and is released into the cytosol, where it associates with Smad4. The pathway can be endogenously inhibited, which prevents the binding of smad2/3 to the receptor by smad6/7 [34]. TGIF2 inhibited phosphorylation of smad2/3, while Hu et al. demonstrated that smad3 mediated TGF-*β*-induced *α*-SMA expression [33]. The binding of TGIF2 and miR-34a promoted smad2/3 phosphorylation which increased the expression of the TGF-*β*1/smad pathway.

We proved that miR-34a bound to the 3′-UTR of TGIF2 by dual-luciferase reporter assay. Three types of TGIF2 3′-UTR mutants were studied (TGIF2 Mut1, TGIF2 Mut2, and TGIF2 Mut3). TGIF2 Mut1 and TGIF2 Mut2 both decreased the relative luciferase activity, while TGIF2 Mut2 decreased more significantly than TGIF2 Mut1. This result demonstrated that miR-34a bound to both sites, but the site at 90-97 may play a more important role in the combining of miR-34a and TGIF2. According to miRBase, the mirSVR scores of the two sites were -0.8259 and -0.1205, which showed the site at 90-97 was more stable and more easily for miR-34a to combine with. The binding selectivity was consistent with our results of the binding site. miR-34a bind to both binding sites. However, miR-34a had a higher probability to bind to the site at 90-97 of TGIF2. After HIBEC transfection by TGIF2 siRNA, T*β*R1, TGF-*β*1, and p-smad2/3 were upregulated. CK19 was downregulated, meanwhile *α*-SMA and collagen I were upregulated. These results were consistent with HIBEC transfected with miR-34a mimics and showed that miR-34a promoted EMT and fibrogenesis of HIBEC through the TGF-*β*1/smad pathway by targeting TGIF2.

IL-17 and IL-6 were also upregulated by miR-34a. Interestingly, these two factors not only had inflammatory functions in PBC. IL-17 promoted EMT of HIBECs in vitro. After the treatment with IL-17A, HIBECs changed into bipolar cells with a fibroblastic morphology. The results of real-time PCR and Western blot analyses demonstrated that IL-17A upregulated the expression of vimentin and downregulated E-cadherin in HIBECs. These results suggest that IL-17A may play an important role in the HIBEC EMT [35]. And IL-6 may stimulate EMT, enhance the migration and proliferation, and inhibit apoptosis of HIBECs, thus delaying cellular senescence. For HIBECs treated with IL-6, the mRNA and protein expressions of mesenchymal markers (fibronectin, vimentin, *α*-SMA, and N-cadherin) increased, while the mRNA and protein expressions of epithelial markers (E-cadherin, ZO-1, *β*-catenin, and CK19) decreased [36]. This indicates that miR-34a may promote EMT by other ways not only through the TGF-*β*1/smad pathway.

In summary, we found that miR-34a was overexpressed in the peripheral blood in patients with PBC. We confirmed that in HIBEC, miR-34a promotes EMT and fibrosis by targeting the TGF-*β*1/smad pathway antagonist TGIF2. Inflammatory factors were also regulated by miR-34a; together, they might play an important role in EMT and fibrosis. This study suggests that miR-34a may be a new marker of fibrosis in PBC. Inhibition of miR-34a may be a promising strategy in treating PBC and improving the prognosis of the disease.

## Data Availability

The data used to support the findings of this study are included within the article.

## Supplementary Materials

Supplementary Materials Supplementary Table 1: sequences of miR-34a mimic, inhibitor, and controls. Supplementary Table 2: the dilutions and companies of antibodies in immune cytochemistry assay. Supplementary Table 3: the human PCR primers of CK19, E-cadherin, ZO-1, laminin 1, vimentin, FSP1, *α*-SMA, collagen I, T*β*R1, TGF-*β*1, TGIF2, IL-6 and IL-17, GAPDH, miR-34a, and U6 were purchased from Tsingke Biological Technology. Supplementary Table 4: dilution ratio and company of the primary antibodies in Western blotting.
