# Supplementary material for: MicroRNA-34a Promotes EMT and Liver Fibrosis in Primary Biliary Cholangitis by Regulating TGF-β1/smad Pathway
Source: J Immunol Res. 2021 Apr 23;2021:6890423. doi: 10.1155/2021/6890423 (PMC8087466; doi:10.1155/2021/6890423)
Supplement: Supplementary Materials — Supplementary Table 1: sequences of miR-34a mimic, inhibitor, and controls. Supplementary Table 2: the dilutions and companies of antibodies in immune cytochemistry assay. Supplementary Table 3: the human PCR primers of CK19, E-cadherin, ZO-1, laminin 1, vimentin, FSP1, α-SMA, collagen I, TβR1, TGF-β1, TGIF2, IL-6 and IL-17, GAPDH, miR-34a, and U6 were purchased from Tsingke Biological Technology. Supplementary Table 4: dilution ratio and company of the primary antibodies in Western blotting. [file 6890423.f1.docx]

1. The sequences of primers used for quantitative RT-PCR

cDNA was generated from 1ug of total RNA per sample using anchored oligo (dT) primers (Tiangen, Beijing). Glyceraldehyde-3-phosphate dehydrogenase (GAPDH) was used as internal control. Analysis of miRNAs was performed by the SYBR Green Master Mix (Vazyme Biotech Co., Nanjing) with U6 as internal control. The human PCR primers of CK19, E-cadherin, ZO-1, laminin 1, vimentin, FSP1, α-SMA, collagen I, TβR1, TGF-β1, TGIF2, IL-6 and IL-17, GAPDH, miR-34a and U6 were purchased from Tsingke Biological Technology (Supplementary Table 3).

1. Dilution ratio and company of the primary antibodies in Western blotting :

The protein expression of CK19, E-cadherin, ZO-1, laminin 1, vimentin, FSP1, α-SMA, collagen I, TβR1, TGF-β1, p-smad2/3, TGIF2, IL-6 and IL-17 was evaluated by immunoblotting and GAPDH was used as internal control. The details of antibodies were listed in Supplementary Table 4.

Supplementary Table 1. Sequences of miR-34a mimic, inhibitor and controls.

| Gene name | sequence |
| --- | --- |
| Hsa-mir-34a-5p mimic | 5’-UGGCAGUGUCUUAGCUGGUUGU-3’  5’-AACCAGCUAAGACACUGCCAUU-3’ |
| Hsa-mir-34a-5p inhibitor | 5’-ACAACCAGCUAAGACACUGCCA-3’ |
| mimics negative control | Forward 5’-UUCUCCGAACGUGUCACGUTT-3’ |
|  | Reverse 5’-ACGUGACACGUUCGGAGAATT-3’ |
| inhibitor negative control | 5’-CAGUACUUUUGUGUAGUACAA-3’ |

Supplementary Table 2. The dilutions and companies of antibodies in immune cytochemistry assay.

| Gene name | Dilution of primary antibody | Company of primary antibody | Dilution of secondary antibody | Company of secondary antibody |
| --- | --- | --- | --- | --- |
| Homo CK19 | 1:100 | Proteintech Group, Wuhan, China | Cy3 goat anti-rabbit 1:100 | Wuhan Boster Biological Technology, Wuhan, China |
| Homo α-SMA | 1:250 | Novus Biologicals, US | Cy3 goat anti-mouse 1:100 | Wuhan Boster Biological Technology, Wuhan, China |
| Homo collagen I | 1:200 | Abcam, UK | Cy3 goat anti-rabbit 1:100 | Wuhan Boster Biological Technology, Wuhan, China |

Supplementary Table 3. Primers used for quantitative RT-PCR.

| Gene name | Primer sequence | |
| --- | --- | --- |
| Hsa-mir-34a-5p | Forward | 5’- TGCGCTGGCAGTGTCTTAGCT -3’ |
|  | loop | 5’-GTCGTATCCAGTGCAGGGTCCGAGGTATTCGCACTGGATACGACACAACCAG -3’ |
| Homo U6 | Forward | 5’-CGCTTCGGCAGCACATATAC-3’ |
|  | Reverse | 5’-AAATATGGAACGCTTCACGA-3’ |
| Homo GAPDH | Forward | 5’- GAGATCAACCCACGGATCAACGACA -3’ |
|  | Reverse | 5’- TCATGCAACAACCCGCCCAAT -3’ |
| Homo CK19 | Forward | 5’- TCCAGATGAGCAGGTCCGAGGTTAC -3’ |
|  | Reverse | 5’- CCTCCGTTTCTGCCAGTGTGTCTT -3’ |
| Homo E-cadherin | Forward | 5’-CGTAGCAGTGACGAATGTGG-3’ |
|  | Reverse | 5’-CTGGGCAGTGTAGGATGTGA-3’ |
| Homo ZO-1 | Forward | 5‘-CTAAGGGAGCACATGGTGAAGGTAA-3’ |
|  | Reverse | 5‘-GTCGGGCAGAACTTGTATATGGTTT-3’ |
| Homo LAMB1 | Forward | 5‘-TCAATGAAGAAGTGGAAGGAATG-3’ |
|  | Reverse | 5‘-TCTGGGTGCTGGTAGTAAAAC-3’ |
| Homo vimentin | Forward | 5’- TGAGTACCGGAGACAGGTGCAG -3’ |
|  | Reverse | 5’- TAGCAGCTTCAACGGCAAAGTTC -3’ |
| Homo FSP-1 | Forward | 5’- CCACAAGTACTCGGGCAAAG -3’ |
|  | Reverse | 5’- TGGGCTGCTTATCTGGGAAG -3’ |
| Homo α-SMA | Forward | 5’- TCATGGTCGGTATGGGTCAG -3’ |
|  | Reverse | 5’- CGTTGTAGAAGGTGTGGTGC -3’ |
| Homo collagen I | Forward | 5’- TGGAGAGGAAGGAAAGCGAG -3’ |
|  | Reverse | 5’- ACCAGCTTCACCAGGAGATC -3’ |
| Homo TβR1 | Forward | 5’-AGTAAGACATGATTCAGCCACA-3’ |
|  | Reverse | 5’-CCCAGAATACTAAGCCCATT-3’ |
| Homo TGFβ1 | Forward | 5’-ACACCAACTATTGCTTCAG-3’ |
|  | Reverse | 5’-TGTCCAGGCTCCAAATG-3’ |
| Homo TGIF2 | Forward | 5’- CAGTGATCTAGGTGAGGACGAAGGC -3’ |
|  | Reverse | 5’- CGGGAAATGGTAAACTGATTAGGGT -3’ |
| Homo IL-6 | Forward | 5’-GGTCCAGTTGCCTTCTCCC-3’ |
|  | Reverse | 5’-GTGCCTCTTTGCTGCTTTC-3’ |
| Homo IL-17 | Forward | 5’-TACAACCGATCCACCTCACCT-3’ |
|  | Reverse | 5’-CCCACGGACACCAGTATCTTC-3’ |

Supplementary Table 4. The dilutions and companies of antibodies in western blotting analysis.

| Gene name | Dilution of primary antibody | Company of primary antibody | Dilution of secondary antibody | Company of secondary antibody |
| --- | --- | --- | --- | --- |
| Homo GAPDH | 1:1000 | Hangzhou Xianzhi Biotechnology, Hangzhou, China | HRP goat anti-rabbit 1:50000 | Wuhan Boster Biological Technology, Wuhan, China |
| Homo CK19 | 1:1000 | Proteintech Group, Wuhan, China | HRP goat anti-rabbit 1:50000 | Wuhan Boster Biological Technology, Wuhan, China |
| Homo E-cadherin | 1:1000 | Affinity Biosciences, Changzhou, China | HRP goat anti-rabbit 1:50000 | Wuhan Boster Biological Technology, Wuhan, China |
| Homo ZO-1 | 1:1000 | Proteintech Group, Wuhan, China | HRP goat anti-rabbit 1:50000 | Wuhan Boster Biological Technology, Wuhan, China |
| Homo laminin 1 | 1:1000 | Affinity Biosciences, Changzhou, China | HRP goat anti-rabbit 1:50000 | Wuhan Boster Biological Technology, Wuhan, China |
| Homo vimentin | 1:1000 | Proteintech Group, Wuhan, China | HRP goat anti-rabbit 1:50000 | Wuhan Boster Biological Technology, Wuhan, China |
| Homo FSP-1 | 1:500 | Proteintech Group, Wuhan, China | HRP goat anti-rabbit 1:50000 | Wuhan Boster Biological Technology, Wuhan, China |
| Homo α-SMA | 1:1000 | Abcam, UK | HRP goat anti-mouse 1:50000 | Wuhan Boster Biological Technology, Wuhan, China |
| Homo collagen I | 1:1000 | Abcam, UK | HRP goat anti-rabbit 1:50000 | Wuhan Boster Biological Technology, Wuhan, China |
| Homo TβR1 | 1:1000 | Abcam, UK | HRP goat anti-rabbit 1:50000 | Wuhan Boster Biological Technology, Wuhan, China |
| Homo TGFβ1 | 1:1000 | Proteintech Group, Wuhan, China | HRP goat anti-rabbit 1:50000 | Wuhan Boster Biological Technology, Wuhan, China |
| Homo p-smad2/3 | 1:500 | Abcam, UK | HRP goat anti-rabbit 1:50000 | Wuhan Boster Biological Technology, Wuhan, China |
| Homo TGIF2 | 1:1000 | Proteintech Group, Wuhan, China | HRP goat anti-rabbit 1:50000 | Wuhan Boster Biological Technology, Wuhan, China |
| Homo IL-6 | 1:1000 | Proteintech Group, Wuhan, China | HRP goat anti-rabbit 1:50000 | Wuhan Boster Biological Technology, Wuhan, China |
| Homo IL-17 | 1:500 | Bioworld, Nanjing, China | HRP goat anti-rabbit 1:50000 | Wuhan Boster Biological Technology, Wuhan, China |
